# Uptake of carbamazepine by rhizomes and endophytic bacteria of *Phragmites australis*

**DOI:** 10.3389/fpls.2015.00083

**Published:** 2015-02-20

**Authors:** Andrés Sauvêtre, Peter Schröder

**Affiliations:** Plant Endophyte Physiology, Research Unit Microbe–Plant Interactions, Helmholtz Zentrum München GmbH, German Research Center for Environmental HealthMunich, Germany

**Keywords:** pharmaceuticals, carbamazepine, common reed, rhizomes, endophytic bacteria, phytoremediation

## Abstract

Carbamazepine is an antiepileptic and mood-stabilizing drug which is used widely in Europe and North America. In the environment, it is found as a persistent and recalcitrant contaminant, being one of the most prominent hazardous pharmaceuticals and personal care products in eﬄuents of wastewater treatment plants. *Phragmites australis* is one of the species with both, the highest potential of detoxification and phytoremediation. It has been used successfully in the treatment of industrial and municipal wastewater. Recently, the identification of endophytic microorganisms from different plant species growing in contaminated sites has provided a list of candidates which could be used as bio-inoculants for bioremediation of difficult compounds. In this study, *Phragmites australis* plants were exposed to 5 mg/L of carbamazepine. After 9 days the plants had removed 90% of the initial concentration. Endophytic bacteria were isolated from these plants and further characterized. Phylogenetic analysis based on 16S rDNA sequencing revealed that the majority of these isolates belong to three groups: Proteobacteria, Actinobacteria, and Bacteroidetes. Carbamazepine uptake and plant growth promoting (PGP) traits were analyzed among the isolates. Ninety percent of the isolates produce indole acetic acid (IAA) and all of them possess at least one of the PGP traits tested. One isolate identified as *Chryseobacterium taeanense* combines good carbamazepine uptake and all of the PGP traits. *Rhizobium daejeonense* can remove carbamazepine and produces 23 μg/mL of IAA. *Diaphorobacter nitroreducens* and *Achromobacter mucicolens* are suitable for carbamazepine removal while both, *Pseudomonas veronii* and *Pseudomonas lini* show high siderophore production and phosphate solubilization. Alone or in combination, these isolates might be applied as inoculates in constructed wetlands in order to enhance the phytoremediation of carbamazepine during wastewater treatment.

## INTRODUCTION

Daughton and Ternes (1999) first described the occurrence and significance of pharmaceuticals and personal care products (PPCPs) in the environment. They postulated the necessity of developing environmental risk assessment strategies for these substances in addition to the traditional priority pollutants (Daughton and Ternes, 1999). Since then, pharmaceuticals in the aquatic environment have raised increasing interest as it is reflected in the growing number of publications related to their fate (Fatta-Kassinos et al., 2011). Although their concentrations may presently be low, their persistence and bioaccumulation will increasingly cause problems for drinking water quality in the future.

Belonging to this group, carbamazepine is one of the most problematic compounds in wastewater treatment plants (WWTPs). Widely used as antiepileptic and mood stabilizer since the 1970s, this pharmaceutical and its metabolites have commonly been detected in sewage and surface water (Ternes, 1998; Heberer et al., 2002; Tixier et al., 2003; Wiegel et al., 2004). The presence of carbamazepine has also been reported in groundwater (Lapworth et al., 2012) and drinking water as well (Huerta-Fontela et al., 2011). In fact, carbamazepine can reach groundwater without degradation during the soil-aquifer passage (Ternes et al., 2007).

Studies considering influent and eﬄuent PPCPs concentration data from over 40 published sources corresponding to more than 40 pilot and full-scale wastewater treatment facilities have listed carbamazepine as one of the most recalcitrant PPCPs to removal via traditional wastewater treatment (Miège et al., 2009; Oulton et al., 2010). In none of these cases, the removal efficiency exceeded 30%. Because of its persistence, carbamazepine has been applied as an indicator for urban influence on water systems (Strauch et al., 2008).

The rising number of pharmaceutical prescriptions for the aging population results in a higher discharge of the medicaments and their metabolites in sewage water. Subsequently, the presence of pharmaceuticals in surface waters has increased in recent years and it has been accepted that this tendency will continue in the near future. Efficient cleaning systems are needed to avoid or at least to reduce the intrusion of these compounds in our surface and groundwater systems, thus maintaining a high level of drinking water quality. The use of constructed wetlands as final polishing step in WWTPs or as sole treatment in small communities can constitute a good solution for recalcitrant compounds (Verlicchi and Zambello, 2014). If not completely, these systems have shown to reduce significantly the concentration of carbamazepine in the final eﬄuent (Conkle et al., 2008; Matamoros et al., 2008, 2009; Park et al., 2009).

Evidence for the uptake of carbamazepine in different plant species is available from current literature (Herklotz et al., 2010; Winker et al., 2010; Wu et al., 2010, 2012; Shenker et al., 2011; Holling et al., 2012; Tanoue et al., 2012). Carbamazepine has been reported to be taken up and translocated into the aerial plant part, where it can be accumulated or metabolized to secondary products (Dordio et al., 2011). Macrophytes are a well suitable for water cleaning because of their ability to grow in flooded conditions and therefore they are generally used in constructed wetlands of water treatment facilities. Among them, *Phragmites australis* has been successfully utilized for phytoextraction of several xenobiotics including pharmaceuticals (Matamoros et al., 2005; Hijosa-Valsero et al., 2010; Carvalho et al., 2012).

In this context it is of high interest, that plant associated bacteria can aid plants to cope with stress resulting from exposure to xenobiotics. In recent years, plant endophytes capable of degrading xenobiotics have been isolated. Most of the studies focus on organic pollutants such as petroleum derivatives, PAHs, TCE, organochlorines, naphthalene, pyrene, or phenolic compounds (Siciliano et al., 2001; Aken et al., 2004; Germaine et al., 2006, 2009; Sheng et al., 2008; Weyens et al., 2009a, 2010; Yousaf et al., 2011; Ho et al., 2012; Kang et al., 2012; Peng et al., 2013). In re-inoculation experiments, some of these strains have improved remediation, favoring the metabolism of these compounds as well as the fitness of the plant (Afzal et al., 2014). However, research on the degradation of pharmaceuticals by endophytic microorganisms is scarce. Only bacteria from activated sludge in a municipal or ligninolytic fungi have been tested for carbamazepine degradation (Santos et al., 2012; Li et al., 2013).

Currently, carbamazepine uptake is thought to proceed via non-selective diffusion across the membrane and posterior translocation inside the plant (Winker et al., 2010). Therefore, the first steps of detoxification could be achieved by the endophytic community living in the roots and rhizomes of these aquatic plants. Our work aims at elucidating the cooperative uptake and degradation mechanisms of carbamazepine in *Phragmites australis* and its endobacteria and tries to give recommendations for its enhanced removal by phytoremediation. The objectives were to determine the uptake of the compound into the plant, to optimize a method for extraction of endophytic bacteria, to characterize the most abundant isolates and to assay their potential role in carbamazepine metabolism and plant growth promotion.

## MATERIALS AND METHODS

### PLANT MATERIAL

*Phragmites australis* plants were supplied from a local grower (Jörg Petrowski, Eschede, Germany). In order to prepare them for the experiments, shoots were cut above the hypocotyl. Root systems (consisting of primary and secondary roots and rhizomes) were carefully washed in tap water to remove any attached soil, placed in individual pots containing perlite and grown in semi-hydroponic conditions at the Helmholtz Zentrum Muenchen, Bavaria, Germany. Greenhouse conditions were set to a light cycle of 16 h day and 8 h night, temperature of 25°C and RH of 60%. Plants were grown in trays (six pots per tray) in modified Hoagland solution. After 8 weeks, when new biomass had developed, plants of uniform size were selected and placed into individual pots containing 2 L of modified Hoagland solution. To study carbamazepine uptake, a stock solution of carbamazepine (Sigma-Aldrich) in ethanol was added to each pot to a final concentration of 5 mg/L (21.16 μM). Controls containing only perlite were set up to investigate photodegradation, volatilization, adsorption on the plastic pot walls, or hydrolysis. Three pots were set up for each of the four exposure times (0, 1, 4, and 9 days) in the greenhouse and each assay consisted of three replicates, arranged in a completely randomized design. Distilled water was added daily to compensate losses by evapotranspiration (Dordio et al., 2011). Samples were taken at 0, 1, 4, and 9 days of exposure and immediately frozen at -20°C.

### CARBAMAZEPINE ANALYSIS

Carbamazepine concentration in plant nutrient solutions was determined by high performance liquid chromatography (HPLC), using a Varian ProStar HPLC system (Varian ProStar 215 solvent delivery module, autosampler Prostar 410). All samples were prepared in triplicate. Plant nutrient solution samples were filtered using 0.45 μm pore size polyvinylidene fluoride filters (Rotilabo, Carl Roth) before injection. Injection volume was 40 μL. The separation was performed on a C18 ProntoSIL Spheribond ODS 2 (5 μM, 125 × 4 mm, Bischoff) column under reversed phase conditions, applying a linear gradient of eluents (buffer A: H_2_O, 0.1% TFA; buffer B: acetonitrile, 0.1% TFA) and a flow rate of 1 mL/min. The gradient started with 5% B for 2.5 min, ramped up to 95% in 15 min, remained at 95% for 3.5 min, and finally ramped down to 5% in 2 min. Carbamazepine was measured at 210 nm in a photodiode array detector (Varian ProStar 335) and identified by comparison of the spectra and retention time of an authentic standard (Sigma–Aldrich). Calibration curves were constructed from a set of carbamazepine standard solutions ranging from 0.25 to 12 mg/L (1.04–50 μM). Chromatograms were analyzed using MS Workstation version 6.9.3 (Varian).

### ISOLATION OF CULTIVABLE ENDOPHYTIC BACTERIA FROM *Phragmites australis* PLANTS EXPOSED TO CARBAMAZEPINE

After treatment with 5 mg/L carbamazepine for 9 days, roots, rhizomes, and stems were collected from different plants, washed with tap water to remove attached perlite and soil particles and mixed subsequently to constitute three independent samples (root, rhizome, and stem). Samples were separately sliced into 2–3 cm pieces, surface sterilized with a 2% NaClO solution for 20 min under orbital shaking (150 rpm) and rinsed three times with sterile water for 1 min. They were then pestled in a glass mortar in 2 mL of sterile water. One milliliter aliquots were ten-fold diluted and 100 μL of every dilution (10^-1^–10^-6^ dilutions) were spread in duplicate onto R2A (Reasoner’s 2A agar) and PDA (potato dextrose agar) plates and incubated at room temperature for 14 days. Carbamazepine was added to the plates at a final concentration of 10 μM. A 100 μL sample of the third rinsing water was plated to verify the efficiency of sterilization. Distinct colony morphotypes were sub-cultured three times to ensure purity and cryopreserved for further characterization.

### DNA EXTRACTION FROM BACTERIAL CULTURES AND AMPLIFICATION OF THE BACTERIAL 16S rRNA

Genomic DNA from bacterial cultures was extracted using the FastDNA^TM^ SPIN kit for soil (MP Biomedicals) according to the recommendations of the manufacturer. Two to 3 mL of fresh cultures were used for this purpose. Universal primers 27FW (5′-AGAGTTTGATCCTGGCTCAG-3′) and 1492 RV were used to partially amplify the 16S rRNA encoding gene from the endophytic bacteria. The 50 μL PCR reaction mixture contained 100–200 ng of extracted DNA, 1 × Top Taq buffer, 200 μM of each dNTP, 200 pM of each primer and 1.25 U of Top Taq polymerase (Qiagen). After initial denaturation at 94°C for 5 min, each thermal cycling consisted of 1 min denaturation at 94°C, 1 min annealing at 52°C and 1.5 min elongation at 72°C. After 35 cycles, a final extension step was performed at 72°C for 10 min. PCR products were separated by electrophoresis on 1% agarose gels. Bands corresponding to approx. 1450 bp were excised and purified using the NucleoSpin® Gel and PCR clean-up kit (Macherey–Nagel) according to the manufacturer’s protocol. The purified PCR products were sequenced using the primers 16S-27f, 16S-609f, 16S-907R, and 16S-1492R in an ABI 3730 48-capillary sequencer (Applied Biosystems). PCR reactions for the sequencing were performed using the BigDye® Terminator v3.1 cycle sequencing kit (Life Technologies) following the instructions of the supplier.

### PHYLOGENETIC ANALYSIS OF *Phragmites australis* ENDOBACTERIA

The identification of phylogenetic neighbors was initially carried out by the BLASTN (Altschul et al., 1997) program against the database containing type strains with validly published prokaryotic names and representatives of uncultured phylotypes (Kim et al., 2012). The top thirty sequences with the highest scores were then selected for calculation of pairwise sequence similarity using global alignment algorithm (Myers and Miller, 1988), which was implemented at the EzTaxon server (; Kim et al., 2012).

The evolutionary history was inferred using the neighbor-joining method (Saitou and Nei, 1987). Phylogenetic analysis was performed using the bootstrap method (2000 replicates; Felsenstein, 2009). Evolutionary distances were computed using the maximum composite likelihood method (MEGA, Tamura et al., 2004). Evolutionary analyses were conducted in MEGA6 (Tamura et al., 2013).

### CARBAMAZEPINE REMOVAL FROM LIQUID CULTURES BY *Phragmites australis* ENDOBACTERIA

Bacterial isolates were tested for carbamazepine removal in liquid cultures. Cells were initially grown from the agar plates in 5 mL of Luria-Bertani (LB) medium until saturation and then 100 μL were transferred to either 5 mL of fresh LB/10 medium containing 50 μM of carbamazepine or 5 mL of fresh AB minimal medium (per liter: 2 g ammonium sulfate, 6 g sodium phosphate dibasic, 3 g potassium phosphate monobasic, 3 g sodium chloride, 1 mL calcium chloride 0.1 M, 1 mL magnesium chloride 1.0 M and 1 mL ferric chloride 0.003 M) containing carbamazepine as sole carbon source (0.4 mM). Controls without inoculum were prepared using 5 mL of the corresponding sterile fresh media supplemented with the corresponding concentration of carbamazepine. All 5 mL prepared cultures and controls were incubated for 5 days at 28°C under orbital shaking (150 rpm). Cultures containing different final cell numbers were then centrifuged at 8000 rpm for 10 min. One milliliter of the supernatant was transferred to a new tube and frozen at -20°C. Carbamazepine concentration was determined by HPLC after thawing each sample and filtering it with a 0.45 μm pore size filter.

### PLANT GROWTH PROMOTION TRAITS DETERMINATION IN ENDOPHYTIC BACTERIA

Phosphate solubilization was determined by the appearance of a clear halo on Pikovskaya’s agar plates after 3 days of incubation at 28°C. Bacterial strains were streaked onto Pikovskaya’s agar medium containing (per liter): 0.5 g yeast extract, 10 g glucose, 5 g calcium phosphate [Ca_3_(PO_4_)_2_], 0.5 g ammonium sulfate [(NH_4_)_2_SO_4_], 0.2 g potassium chloride (KCl), 0.1 g magnesium sulfate (MgSO_4_.7H_2_O), 0.0001 g manganese sulfate (MnSO_4_.H_2_O), 0.0001 g ferrous sulfate (FeSO_4_.7H_2_O) and 15 g agar. Strains that induced a clear zone around the colonies were considered as positive.

Bacterial isolates were assayed for siderophore production on Chrome azurol S agar medium as described by Alexander and Zuberer (1991). Chrome azurol S agar plates were prepared, spot inoculated with bacterial isolates (10 μl of 10^6^ CFU/ml) and incubated at 28°C for 72 h. Development of a yellow–orange halo around the colonies was considered as positive for siderophore production.

Indole acetic acid production was detected according to Bano and Musarrat (2003). The isolates were inoculated in LB medium supplemented with 0.5% glucose and 500 μg/mL tryptophan and incubated at 28°C for 48 h. The cultures were centrifuged at 10000 rpm for 15 min and 2 mL of the supernatant were transferred to a fresh tube to which 100 μL of 10 mM orthophosphoric acid and 4 mL of the Salkowski reagent (1 mL of 0.5 M ferrous chloride in 50 mL of 35% perchloric acid) were added. The mixture was incubated at room temperature for 25 min and the absorbance of pink color developed was read at 530 nm. The IAA concentration in cultures was calculated from a calibration curve of pure IAA (Biochemica) solutions ranging from 10 to 50 μg/mL.

## RESULTS

### CARBAMAZEPINE UPTAKE IN PLANTS

The capacity of *Phragmites australis* to take up carbamazepine was investigated under semihydroponic conditions. Adult plants were grown individually in pots containing perlite and treated with 5 mg/L (21.16 μM) of carbamazepine at *t* = 0. The initial carbamazepine concentration determined by HPLC was 17.84 μM in the control pots and 17.52 μM in the plant samples.

**FIGURE 1:**
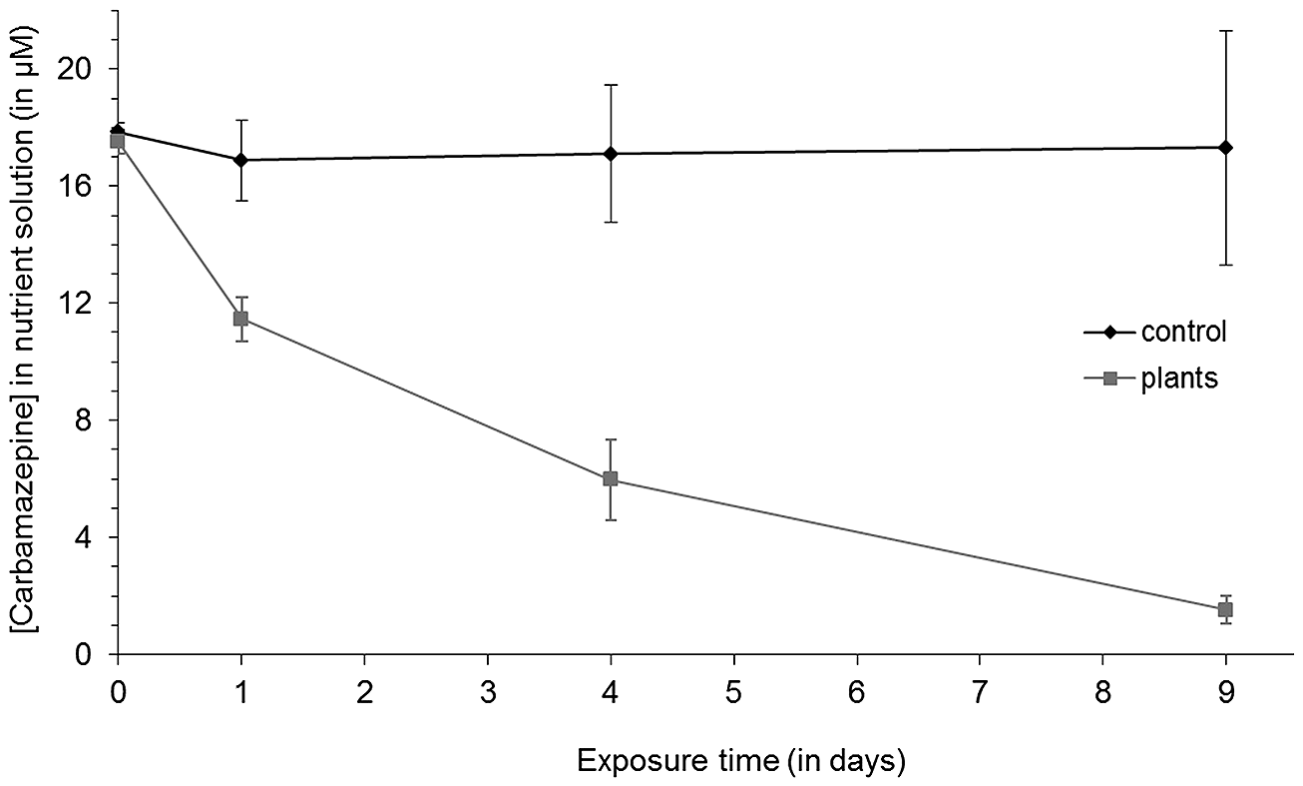
**Carbamazepine removal from the media by *Phragmites australis* at different exposure times.** Vertical error bars represent means ± SD (*n* = 3).

When young reed plants were observed to 5 mg/L carbamazepine in the medium for 9 days, rapid uptake of the substance was observed within the first 24 h (**Figure 1**). Compared to controls without plants, where 5% of the compound was lost, 35% of carbamazepine disappeared from pots with plants. After 4 and 9 days, this initial rate of uptake decreased, but still significant amounts were taken up into the plant. After 4 days 66% were removed, and after 9 days only 10% were left, whereas the concentration in the control pots remained constant. No visual signs of toxicity were detected in *Phragmites australis*. This high uptake led to the assumption that plant and endophyte based mechanisms jointly would favor the removal of the recalcitrant chemical. Therefore, endophytic bacteria were extracted from these exposed plants following standard methods.

### ISOLATION OF ENDOPHYTIC BACTERIA

Extracts were prepared from different plant tissues (stems, roots, and rhizomes) after surface sterilization. No colonies grew on plates inoculated with the last rinsing water, indicating a good efficiency of the sterilization phase. After three subcultures, 41 isolates corresponding to distinct morphotypes were obtained and further characterized. Total DNA extracted from pure cultures was used to amplify bacterial 16S rRNA encoding genes. Sequencing of the fragments allowed taxonomic classification of the morphotypes, and finally 22 redefined distinct species could be identified (**Table 1**).

**Table 1:** Taxonomic identification of endophytic bacteria from *Phragmites australis* plants exposed to carbamazepine based on the 16S rDNA sequence compared to validated type strains sequences deposited in the EzTaxon database.

Isolate	Plant tissue	Closest match	Similarity	Group
Cb35	Roots	*Achromobacter mucicolens* LMG 26685 (HE613446)	100.00	β-Proteobacteria
Cb52	Roots, rhizomes	*Acidovorax radicis* N35 (AFBG01000030)	98.87	β-Proteobacteria
Cb22	Roots	*Acidovorax temperans* CCUG 11779 (AF078766)	99.49	β-Proteobacteria
Cb2	Stems	*Aquabacterium citratiphilum* B4 (AF035050)	98.64	β-Proteobacteria
Cb59	Roots	*Candidatus Rhizobium massilae* 90A (AF531767)	99.14	α-Proteobacteria
Cb69	Roots, rhizomes	*Cedeceadavisae* DSM 4568 (ATDT01000040)	100.00	γ-Proteobacteria
Cb66	Rhizomes	*Chitinophaga sancti* NBRC 15057 (AB078066)	98.87	Bacteroidetes
Cb47	Roots, rhizomes	*Chryseobacterium taeanense* PHA3-4 (AY883416)	98.69	Bacteroidetes
Cb55	Rhizomes	*Diaphorobacter nitroreducens* NA10B (AB064317)	99.86	β-Proteobacteria
Cb56	Rhizomes	*Eiseniicola composti* YC06271 (FJ791048)	98.17	β-Proteobacteria
Cb17	Roots	*Flexibacter aurantiacus* ATCC 23107 (M62792)	99.78	Bacteroidetes
Cb4	Roots	*Kocuria palustris* DSM 11925 (Y16263)	99.93	Actinobacteria
Cb62	Rhizomes	*Leifsonia lichenia* strain 2Sb (AB278552)	100.00	Actinobacteria
Cb46	Roots, rhizomes	*Microvirgula aerodenitrificans* DSM 15089 (JHVK01000054)	99.86	β-Proteobacteria
Cb36	Roots	*Pseudomonas arsenicoxydans* VC-1 (FN645213)	99.70	γ-Proteobacteria
Cb65	Roots, rhizomes	*Pseudomonas corrugata* ATCC 29736 (D84012)	99.86	γ-Proteobacteria
Cb31	Roots	*Pseudomonas lini* CFBP 5737 (AY035996)	100.00	γ-Proteobacteria
Cb49	Rhizomes	*Pseudomonas moorei* RW10 (AM293566)	100.00	γ-Proteobacteria
Cb61	Roots, rhizomes	*Pseudomonas veronii* CIP 104663 (AF064460)	100.00	γ-Proteobacteria
Cb54	Rhizomes	*Rhizobium daejeonense* KCTC 12121 (AY341343)	97.69	α-Proteobacteria
Cb58	Rhizomes	*Rhizobium radiobacter* ATCC 19358 (AJ389904)	100.00	α-Proteobacteria
Cb1	Stems	*Staphylococcus epidermidis* ATCC 14990 (L37605)	99.89	Firmicutes

*Numbers in parentheses represent the sequence accession numbers in GenBank.*

**FIGURE 2:**
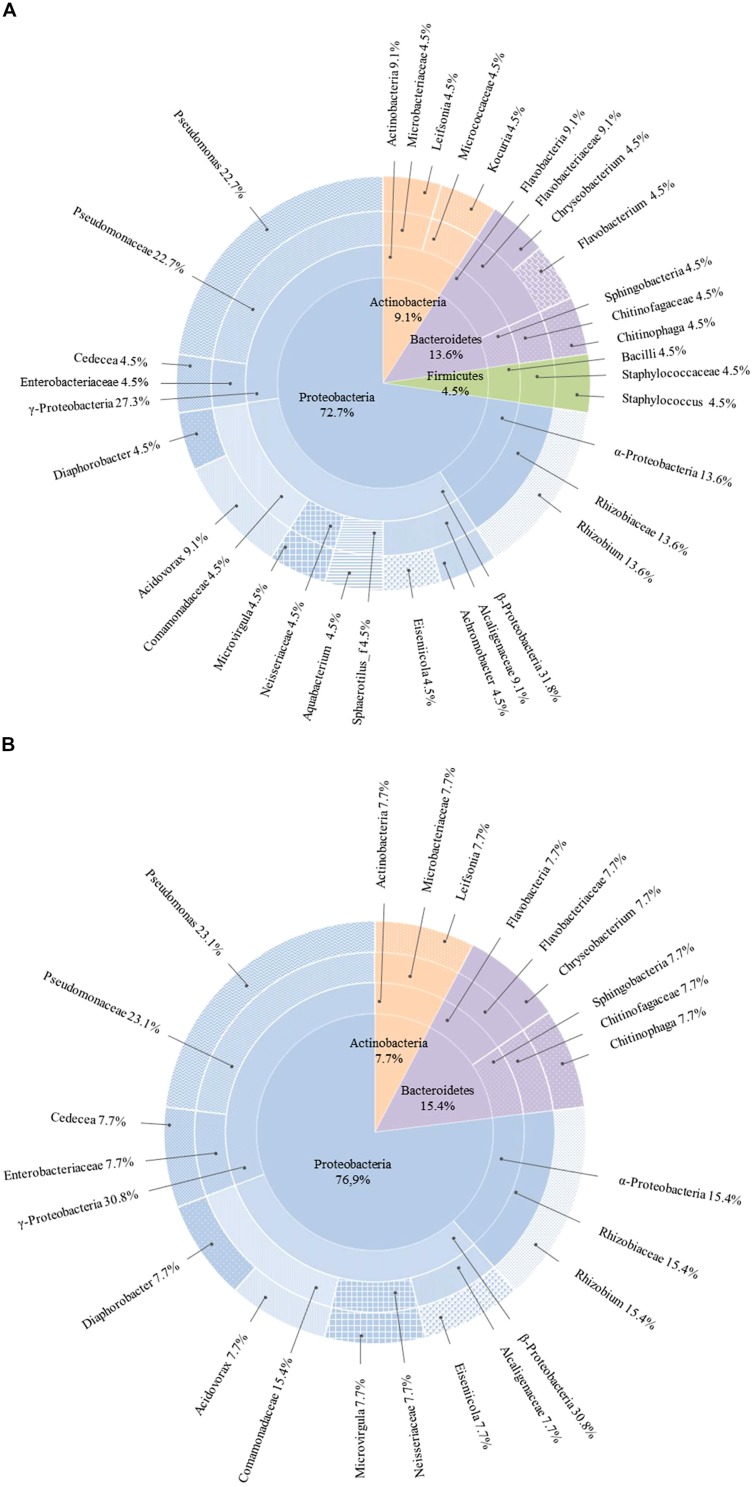
**Taxonomic breakdown of 16S rDNA sequences of total **(A)** and rhizomes **(B)** culturable endophytic bacterial community composition isolated from *Phragmites australis* plants exposed to 5 mg/L of carbamazepine for 9 days.** The central pie shows phylum distribution in percentages and each outer ring breaks progressively down to lowest taxonomic levels (class, family, genera).

**FIGURE 3:**
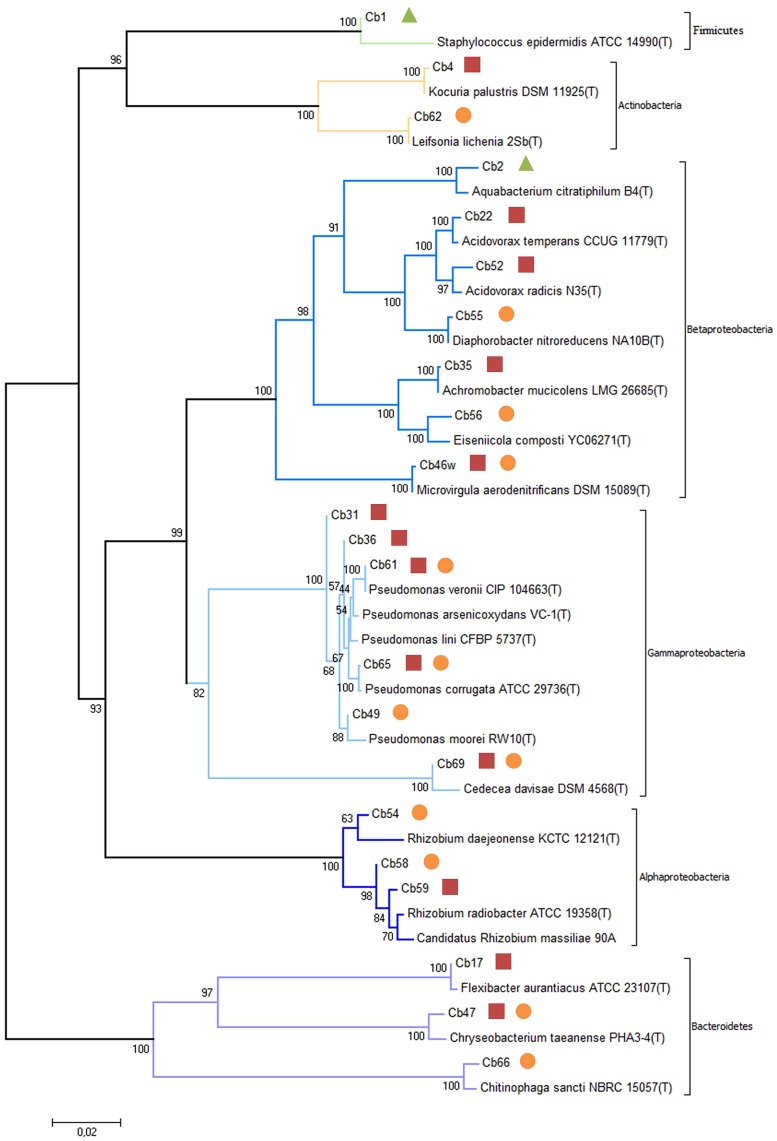
**Evolutionary relationships of isolates from reed plants exposed to carbamazepine based on 16S rDNA sequences obtained from all isolates and closely related sequences from EzTaxon database.** The tree is drawn to scale, with branch lengths in the same units as those of the evolutionary distances used to infer the phylogenetic tree. The percentage of replicate trees in which the associated taxa clustered together in the bootstrap test (2000 replicates) is shown next to the branches. The evolutionary distances were computed using the Maximum Composite Likelihood method. Endophytic bacteria isolated from stems (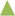), rhizomes (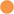) and roots (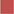).

The diversity of culturable endophytic bacteria was estimated using 16S rDNA sequencing. Phylogenetic analysis of the sequences revealed that the majority of isolates were affiliated with Proteobacteria (72.7%, **Figures 2A and 3**). Other isolates belonged to Bacteroidetes (13.6%), Actinobacteria (9.1%), and Firmicutes (4.5%). The genus *Pseudomonas* (*Gamma-proteobacteria*) was represented by the highest number of identified species (22.7% of the total of isolates) followed by the genus *Rhizobium* (Alphaproteobacteria) with 13.6% and *Acidovorax* (Betaproteobacteria) with 9.1%. Other members belonging to the phylum of Proteobacteria were identified as *Achromobacter*, *Aquabacterium*, *Diaphorobacter*, *Eiseniicola,* and *Microvirgula* (Betaproteobacteria) and *Cedecea* (Gammaproteobacteria) and represented 4.5%. The rest of the isolates (54.6%) were identified as members of different genera of Actinobacteria (*Leifsonia*, *Kocuria*), Bacteroidetes (*Chryseobacterium*, *Flavobacterium,* and *Chitinophaga*), and Firmicutes (*Staphylococcus*), each of them representing 4.5% of the total of isolates.

As far as the endophytic bacteria residing in the rhizomes are concerned, a similar distribution of phyla was found, with 76.9% of the isolates belonging to Proteobacteria, 15.4% to Bacteroidetes and 7.7% to Actinobacteria (**Figure 2B**). No isolates from Firmicutes phylum were present in the rhizomes. Again, the genera *Pseudomonas* and *Rhizobium* represented the highest number of identified species with 23.1 and 15.4%, respectively. The remaining rhizome endobacteria were identified as *Leifsonia* (Actinobacteria), *Chitinophaga,* and *Chryseobacterium* (Bacteroidetes), *Acidovorax*, *Diaphorobacter*, *Eiseniicola,* and *Microvirgula* (Betaproteobacteria) and *Cedecea* (Gammaproteobacteria) with a representation of 7.7%. The genera *Achromobacter*, *Aquabacterium*, *Flavobacterium,* and *Kocuria* were lacking among these isolates.

### CARBAMAZEPINE REMOVAL FROM LIQUID MEDIUM BY ENDOPHYTIC BACTERIA

**FIGURE 4:**
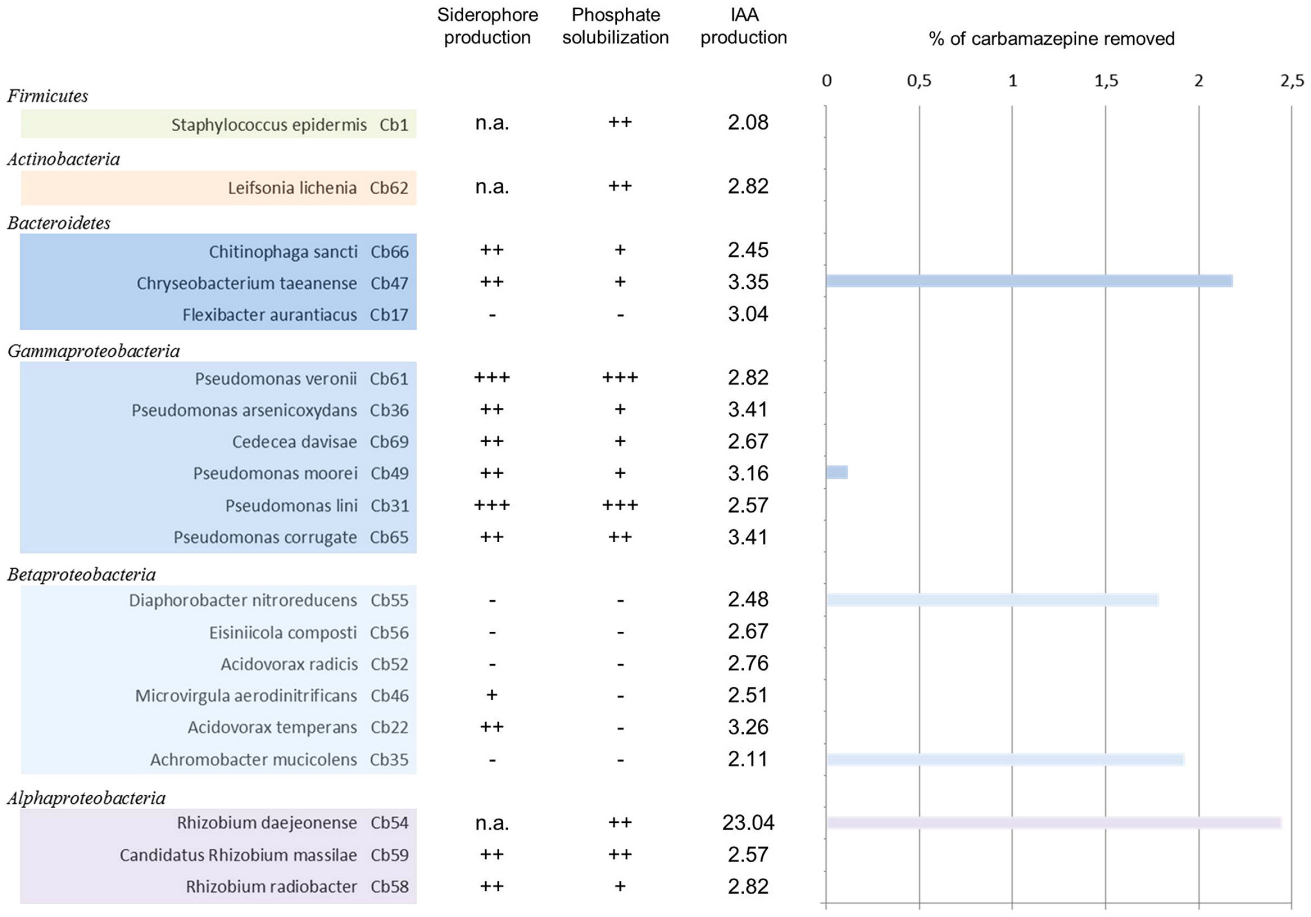
**Carbamazepine removal from liquid cultures by endophytic bacteria associated to *Phragmites australis* and plant growth promoting (PGP) traits.** Bacterial clones are grouped by taxonomic groups (class). Siderophore production and phosphate solubilization are represented by +, ++, or +++. Production of IAA is indicated in μg/mL. The percentage indicates the removal capacity after 5 days in LB/10 medium supplemented with 50 μM carbamazepine.

Bacterial isolates were tested for carbamazepine uptake from liquid media. When strains were grown for 5 days in presence of 50 μM carbamazepine in small liquid LB/10 cultures, slight differences were recorded between the tested strains (**Figure 4**). Sterile controls were incubated under the same conditions to exclude potential effects of photooxidation, volatilization or adsorption to the tube walls on carbamazepine concentration. Only a few strains showed an uptake of the compound with rates ranging from 0.1 to 2.4%. The highest uptake was found within Alphaproteobacteria (*R. daejeonense* with 2.45%), Bacteroidetes (*Chriseobacterium taeanense* with 2.18%), and Betaproteobacteria (*A. mucicolens* with 1.93% and *D. nitroreducens* with 1.78%). One isolate affiliated to *Pseudomonaceae* family (Gammaproteobacteria) showed minor uptake (*Pseudomonas moorei* with 0.12%).

### PLANT GROWTH PROMOTING CHARACTERISTICS

Phosphate solubilization, siderophore production, and auxin production were determined following qualitative or quantitative methods (**Figure 4**). All of the isolates had at least one of the PGP traits tested. Among them, 90% were able to produce indolacetic acid, whereas 59% were able to solubilize mineral phosphate and 54% secreted siderophores into the growth medium. Around 45% of the isolates presented the three PGP traits tested. Most of these isolates belong to Gammaproteobacteria, Alphaproteobacteria, and Bacteroidetes. Whereas 45% were able to produce siderophores and solubilize phosphate, 59% were able to solubilize phosphate and produce IAA.

Indole acetic acid producers were present in all plant tissues, with values in most of the cases ranging between 2.08 and 3.41 μg/mL. All isolates from rhizomes could produce IAA and among them, *R. daejeonense* showed an exceptionally high production of IAA (23.04 μg/mL). Siderophore producers were equally distributed between roots and rhizomes (69 and 67% of root and rhizome isolates, respectively) whereas phosphate solubilizers were predominantly found in rhizomes (75% of rhizomes isolates against 54% of root isolates). None of the Betaproteobacteria isolates could solubilize phosphate.

Ten isolates possessed the three PGP traits tested. This group comprised all members of Gammaproteobacteria (*Pseudomonas veronii*, *Pseudomonas lini*, *Pseudomonas moorei*, *Pseudomonas arsenicoxydans*, *Pseudomonas coorrugata,* and *Cedecea davisae*), two members from Alphaprotebacteria (*R. radiobacter* and *Candidatus R. massilae*) and two members from Bacteroidetes (*Chitinophaga sancti* and *Chriseobacterium taeanense*).

## DISCUSSION

### CARBAMAZEPINE UPTAKE BY COMMON REED

Recently the uptake of carbamazepine into the wetland species *Typha latifolia* has been demonstrated (Dordio et al., 2011). In that paper, it was shown that within 7 days, 28% of a 2 mg/L (corresponding to 8.4 μM) initial concentration had been taken up by the plant. This amount is significantly lower than the uptake rate obtained here with reed at a higher initial concentration (removal of 90% after 9 days). Measurements of carbamazepine remaining in the control pots after 9 days did not reveal any adsorption to the perlite or vessel walls over time. Some studies have shown that the uptake of organic xenobiotics in *Phragmites* is correlated to the log K_OW_ and pK_a_ of the xenobiotic and highest with compounds exhibiting log K_OW_s between 1 and 3 (Schröder et al., 2008). Carbamazepine, with a log K_OW_ of 2.45, is transported faster from roots to shoots in *Phragmites* than in *Typha*. Chazarenc et al. (2010) were not able to attribute any differences in evapotranspiration rates to the morphology of both species. Hence, other effects like plant biomass, ionic interactions, sorption etc. might be responsible for the more effective accumulation of carbamazepine in *Phragmites* tissues.

### ENDOPHYTIC BACTERIA FROM PLANTS EXPOSED TO CARBAMAZEPINE

Once inside the plant, the compound will be distributed in receiving tissues, predominantly the rhizomes. In order to clarify the potential impact of endophytic bacteria on the fate of carbamazepine we isolated extractable endobacteria from exposed *Phragmites* rhizomes and roots. Carbamazepine pressure was always maintained in order to stimulate growth of metabolizers and to identify potential beneficial microbiota among them.

Li et al. (2010) described the endophytic bacterial community in roots of *Phragmites australis* growing in a wetland in Beijing (They could identify 57 different endophytes, affiliated to Proteobacteria (78.9%), Firmicutes (9%), Bacteroidetes (6.6%), Fusobacteria (2.4%), and a few unidentified bacteria (3%). Our results show a similar representation of the Proteobacteria (72.7%), and an inverse tendency in Bacteroidetes (13.6%) and Firmicutes (4.5%) phyla. In addition, we found members affiliated to Actinobacteria. Fusobacteria were not present among our isolates, probably as they are obligatory anaerobic. Surprisingly, of 57 identified strains, only one was represented among our isolates. This result reveals a high speciation of the root microbiome driven by the plant in response to specific external conditions (in this case, a high concentration of carbamazepine). In fact, we do assume now that the plant (species, cultivar, age, health, and developmental stage) is not the only factor influencing microbial communities in the rhizosphere: a multitude of abiotic factors modulate structural and functional diversity of the rhizosphere microbiome, including soil properties, nutrient status, and climatic conditions (Berg et al., 2014). It has also been discussed that external factors imposed via the host plant such as soil, geographic factors, and anthropogenic management drive the overall structure and function of root microbiomes (Gaiero et al., 2013).

*Pseudomonas* (Gammaproteobacteria) was the most abundant genus among our isolates with 22.7% representation. Because of the metabolic diversity in *Pseudomonas*, members of this genus have been used for the remediation of soils contaminated with organic pollutants such as hydrocarbons (Afzal et al., 2011; Andria et al., 2009), TCE (Weyens et al., 2009b, 2010), naphthalene (Germaine et al., 2009) toluene (Weyens et al., 2009b), and the herbicide 2,4-dichlorophenoxyacetic acid (Germaine et al., 2006). Furthermore, *Pseudomonas* has been studied as model for beneficial plant–microbe interaction. In the present study, one of the *Pseudomonas* isolates, *Pseudomonas moorei*, showed a slight carbamazepine removal from the liquid medium. All of the members affiliated to *Pseudomonas* exhibited all PGP traits tested, and two of them in particular. *Pseudomonas linii* is oxidase-positive, and denitrifies (Delorme et al., 2002) and *Pseudomonas veronii* possesses high oxidase and catalase activity, and denitrifies (Elomari et al., 1996). Thus, these isolates could be beneficial partners for the phytoremediation of organic pollutants, especially in constructed wetlands, were the removal of excess nitrogen is needed.

*Rhizobium* (Alphaproteobacteria) was the second genus with high representation (13.6%) in the isolates. *R. meliloti* has been successfully applied in PAH removal observing an ability of this strain to stimulate the rhizosphere degrading microflora (Teng et al., 2011). Among our isolates, *R. daejeonense* showed the highest uptake of carbamazepine with 2.45% and additionally, the highest production of IAA (23.04 μg/mL) and phosphate solubilization. A *R. daejeonense* strain has also been isolated from a cyanide-degrading bioreactor originally inoculated with an activated sludge from a municipal sewage treatment plant in Daejeon, Korea. It possesses catalase and oxidase activity, forms nodules in *M. sativa* and contains a nifH gene encoding a component of the nitrogenase complex (Quan et al., 2005). These characteristics make this strain one of the most promising candidates for inoculation studies.

Betaproteobacteria constitute a group widely represented among the isolates. Members of this class (together with Gammaproteobacteria) are important for plant development as they can oxidize ammonium to nitrite. In this group, *A. xylosoxydans,* and *Burkholderia* sp. have been used for phytoremediation of catechol and phenol (Ho et al., 2012) and toluene (Weyens et al., 2012), respectively. Among our isolates, *A. mucicolens,* and *D. nitroreducens* showed carbamazepine uptake but neither siderophore production nor phosphate solubilization. Nonetheless, *A. mucicolens* strains grow in the presence of 3% NaCl, reduce nitrate, and nitrite, denitrify, exhibit oxidase activity, and can grow anaerobicaly (Vandamme et al., 2013). *D. nitroreducens*, initially isolated from activated sludge, has interesting characteristics for phytoremediation, such as denitrification and catalase positive, and degradation of poly(3-hydroxybutyrate) and poly(3-hydroxybutyrate-co-hydroxyvalerate) under aerobic and anaerobic denitrifying conditions (Khan and Hiraishi, 2002).

Bacteroidetes represented the second most abundant phylum with 13.6% of the isolates. *Chryseobacterium taeanense* is the best strain in this group as reveals the presence of the three PGP traits tested and the uptake of carbamazepine. In terms of tissue compartmentation, there seems to be no strong difference between rhizomes and roots except for the absence of Firmicutes in rhizomes and a higher presence of phosphate solubilizers.

### METABOLISM OF CARBAMAZEPINE

In summary, of 22 identified and isolated endophytic bacteria, seven isolates were chosen as best candidates for further inoculation studies. Out of these seven, three have both, carbamazepine degradation, and PGP properties (*R. daejeonense*, *Chryseobacterium taeanense,* and *Pseudomonas moorei*), two exhibit strong PGP traits (*Pseudomonas veronii* and *Pseudomonas lini*) and two showed carbamazepine uptake but no PGP traits (*D. nitroreducens* and *A. mucicolens*). The relatively low rates of microbial carbamazepine uptake are in accordance with research published so far. Regarding microbial degradation studies, carbamazepine remains a notorious compound for its poor elimination, never showing removal levels higher than 30% across 20 studies examining lab and full scale subsurface flow, lab scale sequencing batch reactor, lab scale anaerobic digester, pilot, and lab scale membrane bioreactor, and full, pilot, and lab scale WWTP systems (Onesios et al., 2009). We believe that the role of plants may be decisive in this respect, and that cooperation between plants and associated bacteria might be necessary to boost elimination of problematic compounds such as carbamazepine. Plant transpiration will aid to enhance uptake of those compounds from the media, and endophytic bacteria will perform the first steps in detoxification, before typical plant detoxification mechanisms take over and convert the xenobiotic into non-toxic breakdown products. Either alone or in combination, the application of these strains to *Phragmites australis* or to other plant species could improve the plant’s fitness in xenobiotic stress conditions and contribute to carbamazepine removal in constructed wetlands. Nevertheless, further inoculation studies need to be performed before implementation in WWTPs.

## CONCLUSION

In this study, we have isolated endophytic bacteria from plants treated with 5 mg/L carbamazepine, a concentration 20–80 times higher than those usually found in municipal sewage water. Plants were able to remove 90% of the initial concentration from nutrient media within 9 days. The cultivable microbial community was characterized by sequencing of 16S rRNAgene amplicons. Its degrading capacity was investigated in both diluted nutrient and minimal media. Plant growth promoting (PGP) traits such as phosphate solubilization, siderophore, and IAA production were analyzed. We found some interesting degrader isolates as well as some which contribute to the fitness of the plants under stress. These strains could serve in the future to improve *in planta* degradation of the antiepileptic drug in wetland-based waste water treatment plants. Further studies on this fruitful cooperation between plants and their endophytes are essential for an application at field scale.

## Conflict of Interest Statement

The authors declare that the research was conducted in the absence of any commercial or financial relationships that could be construed as a potential conflict of interest.
